# Gold nanomakura: nanoarchitectonics and their photothermal response in association with carrageenan hydrogels

**DOI:** 10.3762/bjnano.15.56

**Published:** 2024-06-07

**Authors:** Nabojit Das, Vikas, Akash Kumar, Sanjeev Soni, Raja Gopal Rayavarapu

**Affiliations:** 1 Nanomaterial Toxicology Laboratory, Drug and Chemical Toxicology Group, Food, Drug & Chemical, Environment and Systems Toxicology (FEST) Division, CSIR-Indian Institute of Toxicology Research (CSIR-IITR), Vishvigyan Bhawan, 31 Mahatma Gandhi Marg, Lucknow 226001, India; 2 Academy of Scientific and Innovative Research (AcSIR), Ghaziabad 201002, India; 3 Biomedical Applications Group, CSIR-Central Scientific Instruments Organisation, Sector 30C, Chandigarh 160030, India

**Keywords:** anisotropy, hydrogel, kappa-carrageenan, metal nanoparticles, nanoarchitectonics, nanomakura, photothermal properties, surfactants

## Abstract

Photothermal conversion of light into heat energy is an intrinsic optical property of metal nanoparticles when irradiated using near-infrared radiation. However, the impact of size and shape on the photothermal behaviour of gold nanomakura particles possessing optical absorption within 600–700 nm as well as on incorporation in hydrogels is not well reported. In this study, nanomakura-shaped anisotropic gold nanoparticles (AuNMs) were synthesized via a surfactant-assisted seed-mediated protocol. Quaternary cationic surfactants having variable carbon tail length (*n* = 16, 14, 12) were used as capping for tuning the plasmon peak of gold nanomakura within a 600–700 nm wavelength. The aspect ratio as well as anisotropy of synthesized gold nanomakura can influence photothermal response upon near-infrared irradiation. The role of carbon tail length was evident via absorption peaks obtained from longitudinal surface plasmon resonance analysis at 670, 650, and 630 nm in CTAB-AuNM, MTAB-AuNM, and DTAB-AuNM, respectively. Furthermore, the impact of morphology and surrounding milieu of the synthesized nanomakuras on photothermal conversion is investigated owing to their retention of plasmonic stability. Interestingly, we found that photothermal conversion was exclusively assigned to morphological features (i.e., nanoparticles of higher aspect ratio showed higher temperature change and vice versa irrespective of the surfactant used). To enable biofunctionality and stability, we used kappa-carrageenan- (k-CG) based hydrogels for incorporating the nanomakuras and further assessed their photothermal response. Nanomakura particles in association with k-CG were also able to show photothermal conversion, depicting their ability to interact with light without hindrance. The CTAB-AuNM, MTAB-AuNM, and DTAB-AuNM after incorporation into hydrogel beads attained up to ≈17.2, ≈17.2, and ≈15.7 °C, respectively. On the other hand, gold nanorods after incorporation into k-CG did not yield much photothermal response as compared to that of AuNMs. The results showed a promising platform to utilize nanomakura particles along with kappa-carrageenan hydrogels for enabling usage on nanophotonic, photothermal, and bio-imaging applications.

## Introduction

Nanoarchitectonics is the fabrication of functional material systems by architecting atoms, molecules, and nanomaterials as building blocks [1]. This interplay at the nanoscale renders a plethora of unique physicochemical properties to nanomaterials. These unique properties are due to the mean free path of an electron in a metal which is ≈10–100 nm at room temperature [2]. The mean free path of an electron is influenced by shape/size of the metal nanoparticles which ultimately governs their physical, chemical, optical, magnetic, catalytic, and electronic properties [3]. All plasmonic metals exhibit optical phenomena over a range of electromagnetic radiation. Plasmon resonance in spherical nanoparticles can be stretched over a relatively small wavelength range by changing the diameter, whereas casting anisotropy serves an extra degree of freedom for controlling the plasmon band over a range of visible to infrared (IR) spectrum [4]. Gold nanoparticles are well-known noble metal materials whose resonance occurs in both visible and infrared range of the electromagnetic spectrum, rendering pertinence in various disciplines such as surface-enhanced Raman scattering (SERS), optical sensors, fluorescence (SPR) sensor chips, deoxyribonucleic acid (DNA) sensors, electrochemical sensors, and photothermal therapy (PTT) [5]. These technologies are based on the principle of elastic scattering properties of metal nanoparticles and on the shift in the plasmon band, which is more prominent in the case of anisotropic nanoparticles in comparison to isotropic ones [6]. Several anisotropic gold nanoparticles (AuNPs) have been synthesized and studied in recent years including nanorings, nanoprisms, nanoplates, crooked nanorods, dendrimers, nanocubes, nanodumbells, dogbones, and nanomakura [7–14]. Vis-à-vis to spherical nanoparticles, anisotropic nanoparticles do not require aggregation to generate hotspots due to readily obtained tunability of higher aspect ratios during synthesis [13]. Engineering of anisotropic AuNPs with controlled aspect ratio has therefore become an emerging topic for research.

Anisotropic gold nanoparticles due to their ability to absorb NIR/IR wavelengths show efficient heat-generating capability. It is due to the strong absorption and scattering of light from visible to NIR/IR region that has brought radical advancement in the field of biomedical sciences. The manifestation of longitudinal surface plasmon resonance (LSPR) at the NIR/IR region enhances the efficiency of light absorption and scattering, which is a million times more intense than that of conventionally used organic dyes [15]. The NIR/IR wavelengths have the ability to penetrate biological tissues without using invasive modalities for diagnostic or therapeutic purposes. Also, gold nanoparticles possess an intrinsic capacity to liberate heat upon irradiation of NIR/IR lasers making them a suitable candidate for photothermal therapy (PTT). Previous studies reported morphology-dependent heat liberation in gold nanoparticles where anisotropic particles (gold nanorods) showed ≈5 times faster heat generation as compared to that of spherical nanoparticles [16]. However, localized surface plasmon resonance of anisotropic gold nanoparticles are also susceptible to its surrounding medium, thus impacting photothermal effects of these particles in different media [17]. A very recent study reported photothermal effect of gold nanorods of shorter and longer lengths in gel and aqueous media [18].

In recent times, anisotropic nanoparticles due to their unique physicochemical properties have garnered attention among researchers and enabled them to manoeuvre into an aspiring venture of soft intelligent materials [19]. Soft intelligent materials are primarily hydrogel materials that are stimuli responsive. A recent study showed the use of hydrogels by incorporating gold nanorods for the development of thermo-responsive actuators [20]. The photothermal conversion of gold nanorods is due to their ability to absorb light in the NIR/IR region, ultimately leading to thermoresponsive performance of hydrogel actuators. A concise review on several reports also discussed the potential application of gold nanoparticles in developing hydrogel actuators/sensors for biomedical soft materials [21]. Similarly, a very recent introduction of kappa-carrageenan (k-CG) into the field of nanotechnology has shown immense potential in the synthesis of plasmonic metal nanoparticles [22]. K-carrageenan is well known and is being used in several biomedical applications due to its excellent biocompatibility. However, its thermoresponsive behaviour in association with anisotropic gold nanoparticles is yet to be explored towards photothermal response [23].

In the present work, we developed pillow-shaped (called nanomakura in Japanese) AuNPs using surfactants myristyltrimethylammonium bromide (MTAB, C_14_) and dodecyltrimethylammonium bromide (DTAB, C_12_) with carbon tails containing 14 and 12 carbon atoms, respectively, other than hexadecyltrimethylammonium bromide (CTAB, C_16_), which has 16 carbon atoms in the carbon tail via seed-mediated growth method described elsewhere [24]. We examined the effect of different carbon tail lengths of these surfactants in determining nanoparticle morphology (aspect ratio) and photothermal response. The growth solution used was kept uniform in case of all three surfactants to establish the specific effect of carbon tail length on the respective aspect ratios of the nanoparticles. The synthesis of gold nanomakura particles via seed-mediated growth has been discussed elsewhere [12]. However, the effect of different carbon tail lengths on optical, structural, chemical properties, and photothermal response has been established in the present work. Also, the photothermal responses of the nanoparticles in association with k-CG hydrogels were assessed, indicating their potential in biomedical applications. Furthermore, characterization such as zeta potential, crystallinity, and surface functionalization were investigated through ZetaSizer, X-ray diffraction (XRD), and FTIR, respectively, to establish physicochemical properties of the synthesized nanomaterials.

## Results

### Synthesis, optical spectroscopy, and zeta potential

**Figure 1:**
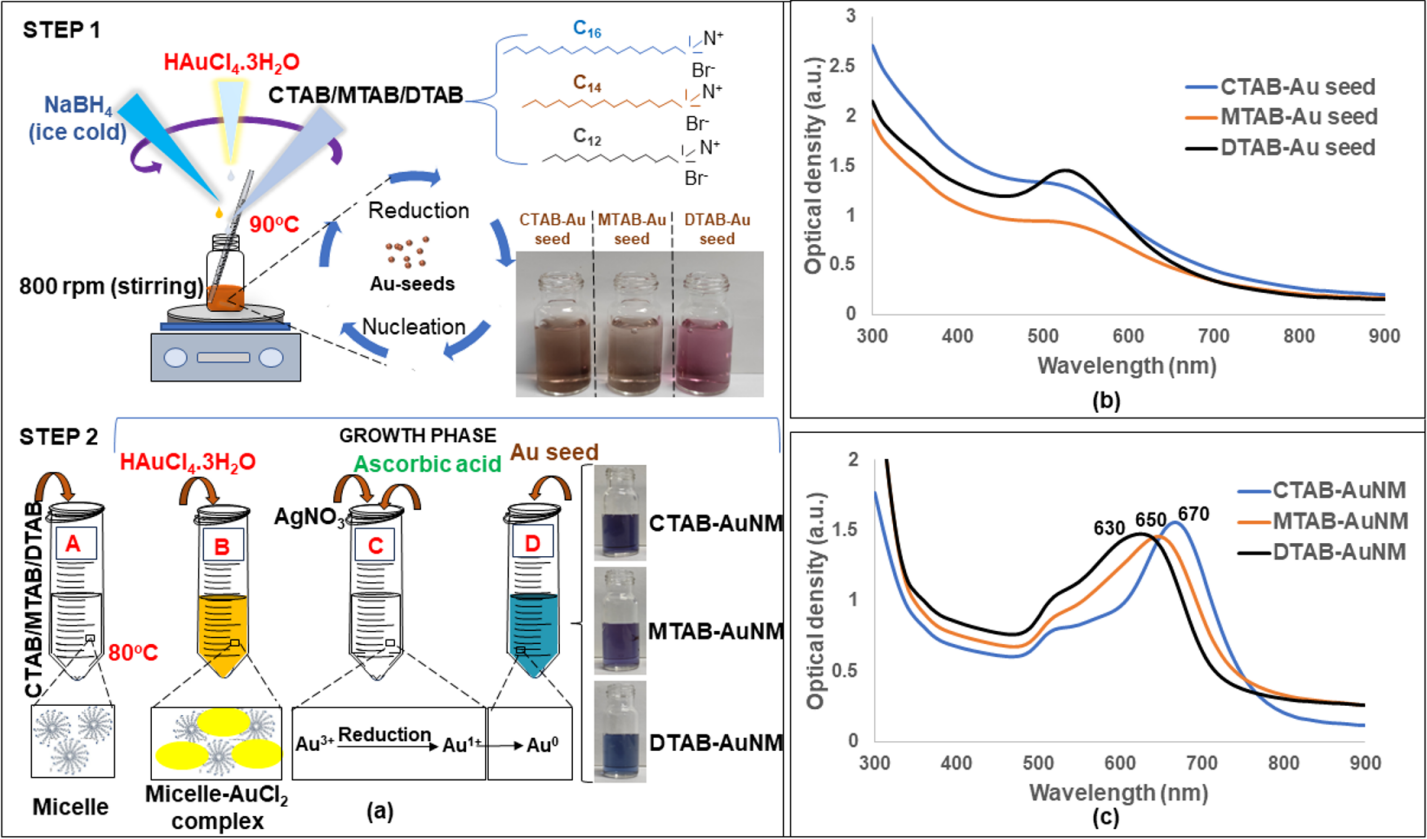
(a) Seed-mediated synthesis of CTAB-AuNM, MTAB-AuNM, and DTAB-AuNM, a two-step reduction method showing gold (Au) seed preparation and formation of AuNMs in growth medium. (b) Absorption spectra of different surfactant-capped Au seeds over a wavelength range of 300–900 nm. (c) Absorption spectra of CTAB-AuNM, MTAB-AuNM, and DTAB-AuNM, respectively, over a wavelength range 300–900 nm.

Anisotropic gold nanoparticles of makura shape were synthesized using seed-mediated approach as shown in Figure 1a. The Au seeds were prepared as shown in step 1 and were used subsequently in step 2 for the preparation of AuNMs. Figure 1b shows the absorption spectra of Au seed capped with different surfactants. Absorption spectra of CTAB-AuNM, MTAB-AuNM, and DTAB-AuNM in the range of visible to NIR are shown in Figure 1c.

The absorption spectra of the synthesized nanoparticles exhibited multimodal SPR peaks, namely transverse surface plasmon resonance (TSPR) and longitudinal surface plasmon resonance (LSPR) respectively. The TSPR and LSPR peaks were observed in the electromagnetic region of 520–530 nm and 600–700 nm, respectively. The LSPR peak obtained at the NIR region in the case of CTAB-, MTAB-, and DTAB-capped gold nanomakuras were 670, 650, and 630 nm, respectively. The absorption spectra exhibited at the visible/NIR region clearly indicated the descending order of the long axes (LSPR) of the nanoparticles in this manner: CTAB-AuNM > MTAB-AuNM > DTAB-AuNM. However, the inflection point at 520–530 nm is similar for all three nanoparticle types with different capping. Table 1 clearly shows the positive zeta potential indicating a positive charge on the hydrodynamic surface which is well known in the case of surfactant-capped gold nanoparticles. The zeta potential of synthesized gold nanoparticles capped with CTAB, MTAB, and DTAB was found to be greater than +30 mV, which strongly confirms their colloidal stability as shown in the Supporting Information File 1, Figure S1.

**Table 1:** Zeta potential values of CTAB-AuNM, MTAB-AuNM, and DTAB-AuNM, respectively.

Sample	Zeta potential (mV)

CTAB-AuNM	+39.2 ± 11.2
MTAB-AuNM	+46.9 ± 25.3
DTAB-AuNM	+35.6 ± 8.69

### Stability of AuNMs in different media

**Figure 2:**
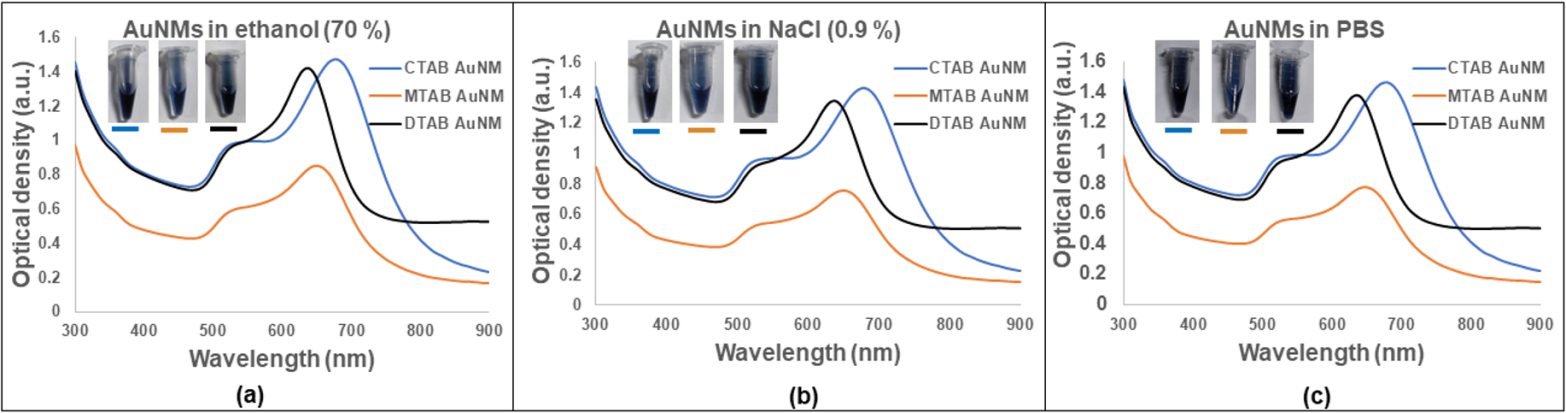
Stability of AuNMs (CTAB-AuNM, MTAB-AuNM, and DTAB AuNM) in different media at the ratio (NPs:media) 3:1 where (a), (b), and (c) represent the stability of AuNMs in ethanol (70%), NaCl (0.9%), and 1X phosphate buffer saline (PBS).

To investigate the stability of the synthesized nanomakuras, different dispersion media were used. The relevant media for biological applications were used for the stability assessment of the nanoparticles. Figure 2a–c shows colloidal stability of AuNMs in ethanol (70%), NaCl (0.9%), and PBS (1X), respectively. The results indicate the colloidal stability of the synthesized AuNMs.

### Stability of AuNMs in k-CG hydrogels

**Figure 3:**
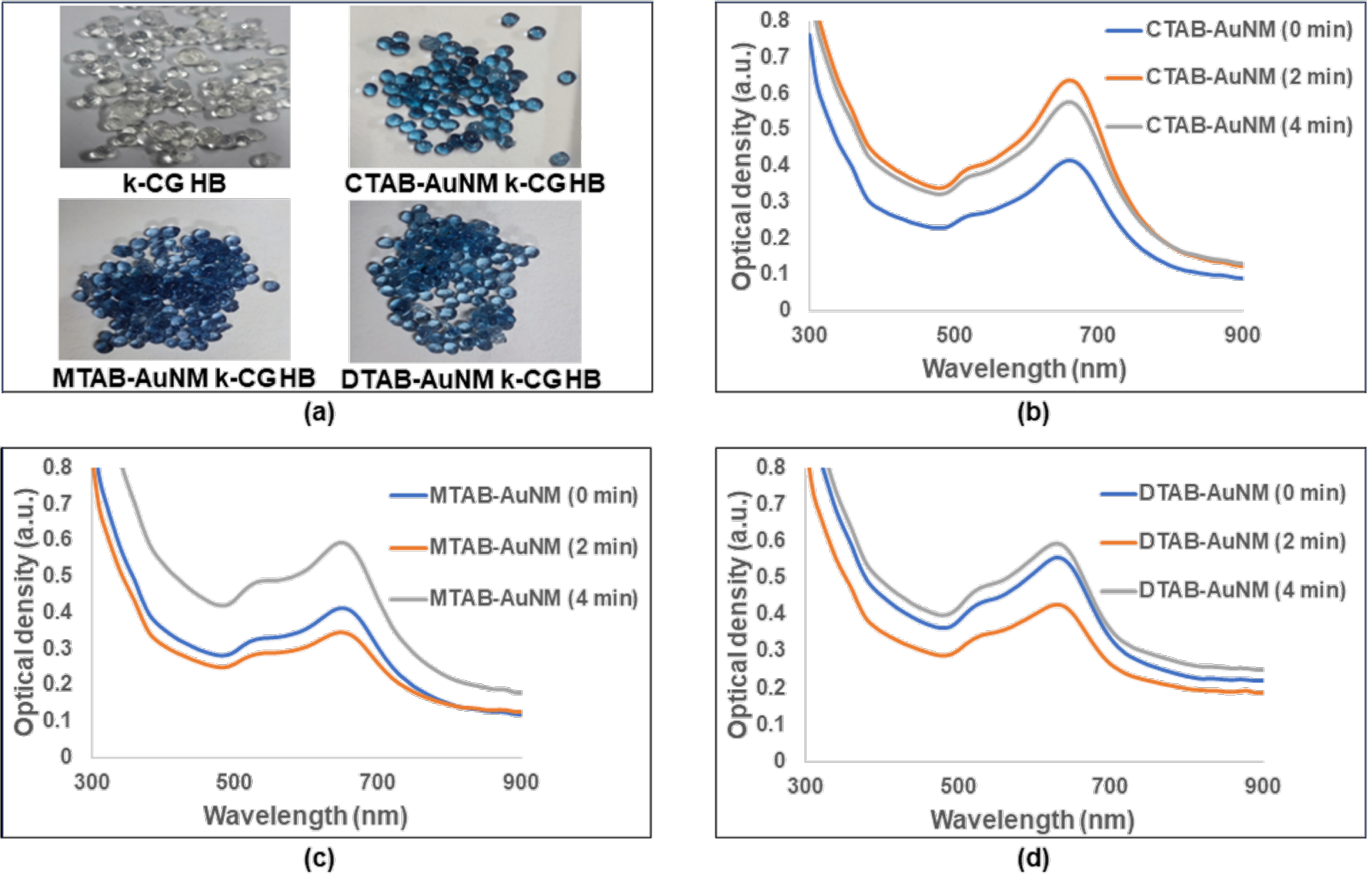
(a) k-CG hydrogel beads (HB) without AuNMs and with incorporated AuNMs; (b), (c), and (d) show stable absorption spectra of CTAB-AuNM, MTAB-AuNM, and DTAB-AuNM in k-CG hydrogels at different time intervals.

The absorption spectral study of AuNMs with different capping, when incorporated in k-CG hydrogels, was essential for their stability assessment. In Figure 3a, the brightly blue coloured hydrogel beads indicated the incorporation of colloidal AuNMs, whereas colourless beads showed no incorporation of nanoparticles. The stability of the AuNMs in k-CG hydrogels was assessed through optical spectrophotometry as shown in Figure 3b–d.

Time-dependent absorption spectra were recorded to ascertain any changes that might occur as the gels solidify. The absorption spectra were measured at 2 min intervals for a total duration of 4 min when the hydrogels completely solidified. We observed no major difference in the absorption spectrum in all three types of AuNMs (i.e., CTAB-AuNM, MTAB-AuNM, and DTAB-AuNM, respectively). However, a temporal change in OD was observed probably due to a change in the refractive index upon solidification of the hydrogels. Therefore, the result clearly showed stable absorption spectra of AuNMs having TSPR and LSPR at visible and NIR regions, respectively, even after their incorporation into k-CG hydrogel beads.

### Transmission electron microscopy and atomic force microscopy measurements

**Figure 4:**
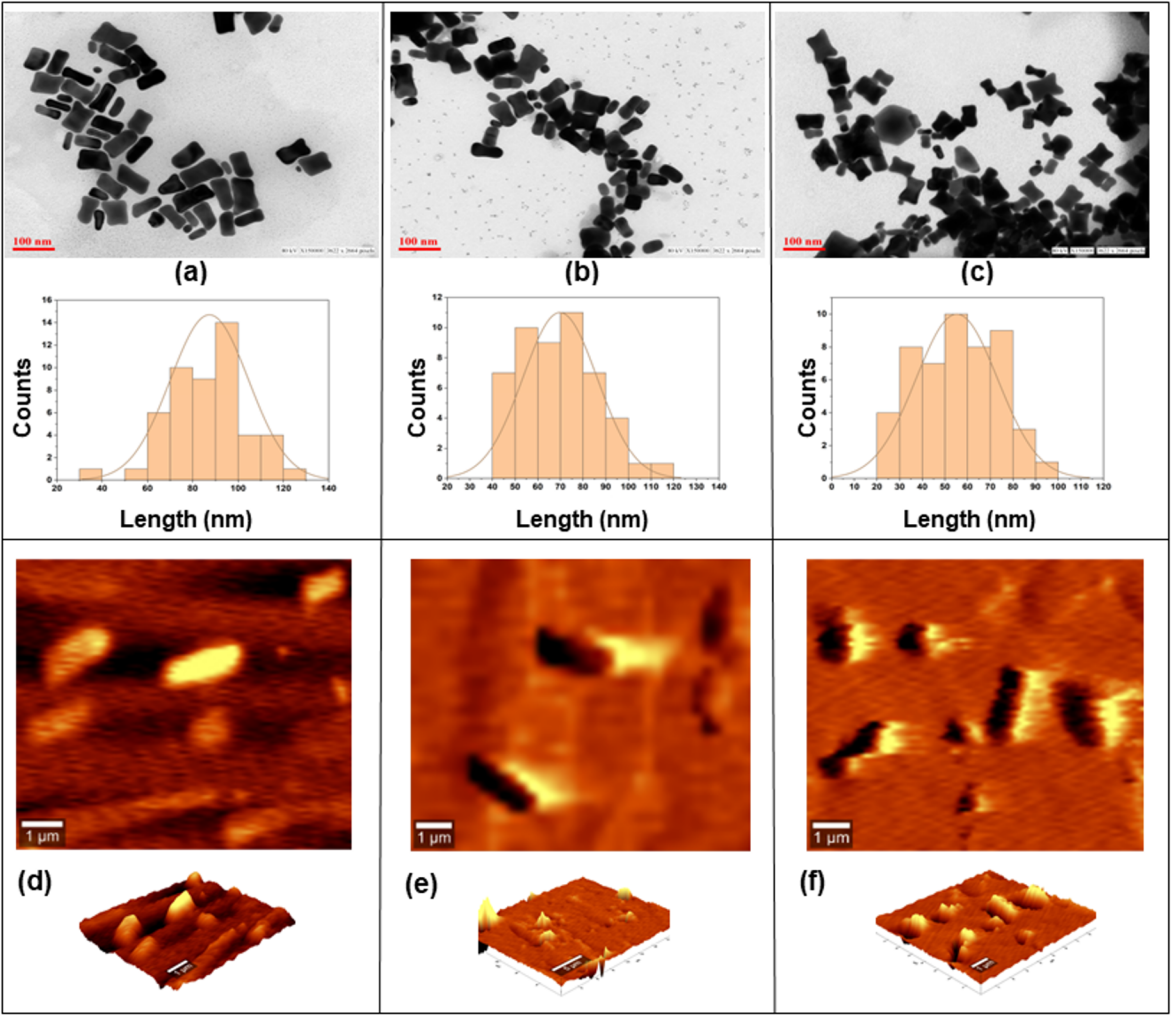
TEM micrographs along with histograms showing morphology and size (length) of (a) CTAB-AuNM, (b) MTAB-AuNM, and (c) DTAB-AuNM, respectively; (d), (e), and (f) showed AFM images of CTAB-AuNM, MTAB-AuNM, and DTAB-AuNM, respectively, along with their 3D topography.

The actual mean size of the synthesized makura-shaped nanoparticles was calculated in terms of length/width aspect ratio. Figure 4 shows transmission electron microscopy (TEM) and atomic force microscopy (AFM) micrographs of CTAB-AuNM, MTAB-AuNM, and DTAB-AuNM, respectively. A total number of 50 nanoparticles were considered for the aspect ratio measurement as shown in Table 2. The analysis was performed using the ImageJ software (NIH, USA). Figure 4d–f shows AFM images of the AuNMs along with their 3D topography, which were performed to confirm the nanomakura shape from a three-dimensional view. Table 2 represents average length, width, and aspect ratio of the nanoparticles where DTAB-AuNM showed the lowest aspect ratio of 1.73. CTAB-AuNM and MTAB-AuNM showed aspect ratios of 2.34 and 1.98, respectively. The observed trend in decreasing aspect ratio is CTAB-AuNM > MTAB-AuNM > DTAB-AuNM.

**Table 2:** Aspect ratio (length:width) of CTAB-AuNM, MTAB-AuNM, and DTAB-AuNM.

Sample	Length (nm)	Width (nm)	Aspect ratio with SE

CTAB-AuNM	87.33	39.76	2.34 ± 0.096
MTAB-AuNM	69.80	36.99	1.98 ± 0.072
DTAB-AuNM	55.22	33.03	1.73 ± 0.049

### Fourier-transform infrared spectroscopy and X-ray diffraction analysis

**Figure 5:**
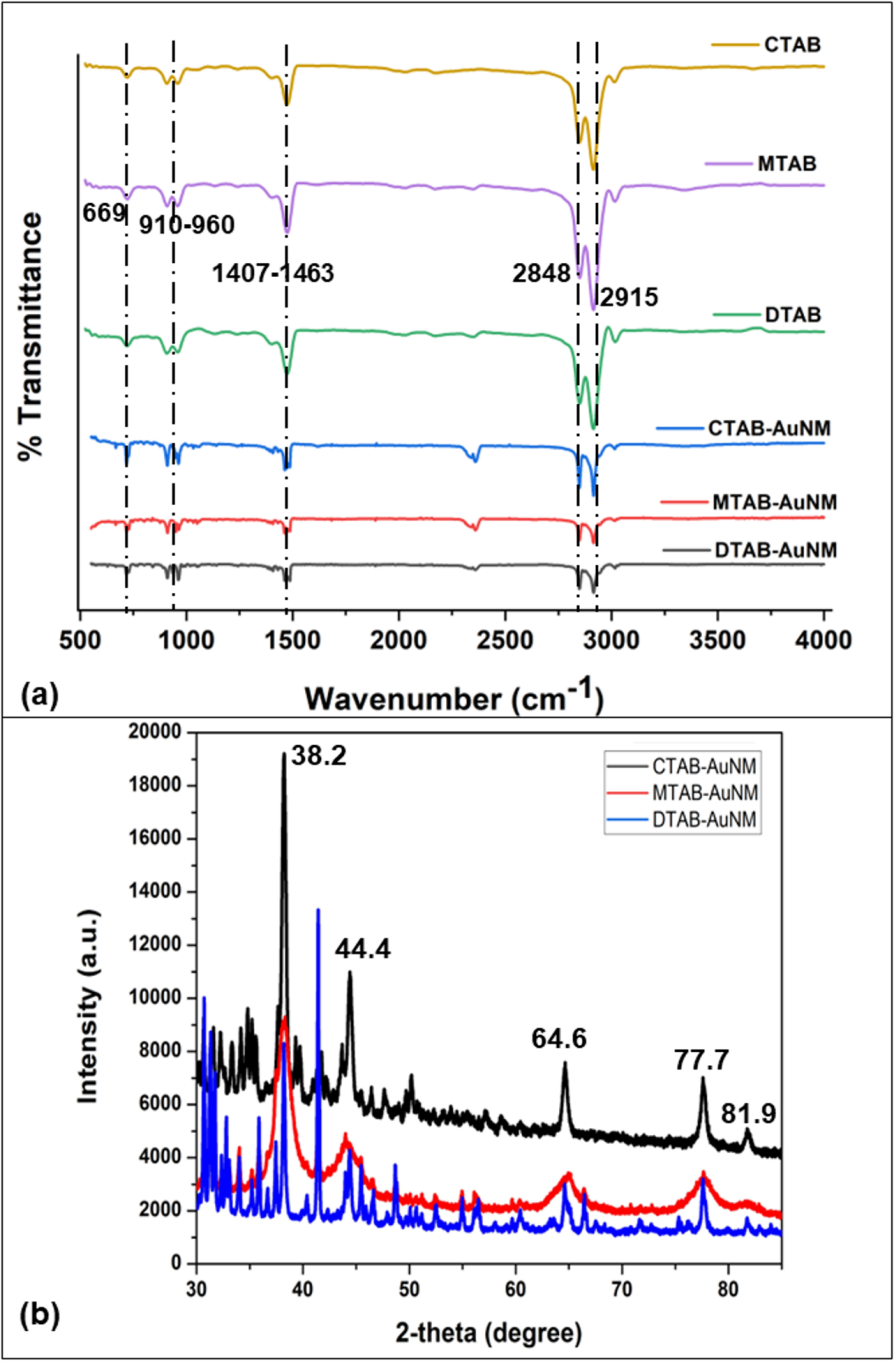
(a) FTIR spectra of CTAB-AuNM, MTAB-AuNM, and DTAB-AuNM, respectively. (b) XRD diffractogram showing diffraction peaks of CTAB-AuNM, MTAB-AuNM, and DTAB-AuNM, respectively.

The CTAB-, MTAB-, and DTAB-capped gold nanoparticles exhibited strong peaks at 2848 and 2915 cm^−1^ due to symmetric and asymmetric C–H stretching, respectively, as well as C–H scissoring of H_3_C–N^+^ at 1407–1463 cm^−1^. The peaks around 910–960 cm^−1^ featured C–N stretching of the surfactants as well as the bending of four CH_2_ groups at 718 cm^−1^. The C–H scissoring vibration of H_3_C–N^+^ and C–N^+^ stretching is relatively less intense and slightly shifted in the case of surfactant-capped gold nanoparticles when compared to the FTIR spectra of pure surfactants (CTAB, MTAB, and DTAB). Pure surfactants (CTAB, MTAB, and DTAB) generally show two bands at 719 and 731 cm^−1^, whereas surfactant-capped gold nanoparticles only show one band at 669 cm^−1^ as shown in Figure 5a [25]. We also observed no peaks in the region of (1500–2000) cm^−1^, which suggested that there were no double bonds formed such as C=C, C=O and C=N.

The crystallinity of synthesized anisotropic gold nanoparticles with surfactants of different carbon tail lengths was explored using XRD as shown in Figure 5b. The diffraction peaks exhibited at 2θ values are 38.2°, 44.4°, 64.6°, 77.7°, and 81.9^o^ which correspond to standard Bragg reflections {111}, {200}, {220}, {311}, and {222}, respectively, of the face-centred cubic (FCC) lattice. The obtained peaks perfectly matched the face-centred cubic lattice structure of metallic gold [26]. The peaks are also consistent with the Joint Committee on Powder Diffraction Standard (JCPDS 04-0784) [27]. Among the obtained diffraction peaks, the peak at 38.2° exhibited maximum intensity. The composition of gold was also confirmed through scanning electron microscopy with energy-dispersive X-ray spectroscopy (SEM/EDX) of the powdered form of AuNM, as shown in Supporting Information File 1, Figure S4.

### Growth mechanism in AuNMs during seed-mediated synthesis

**Figure 6:**
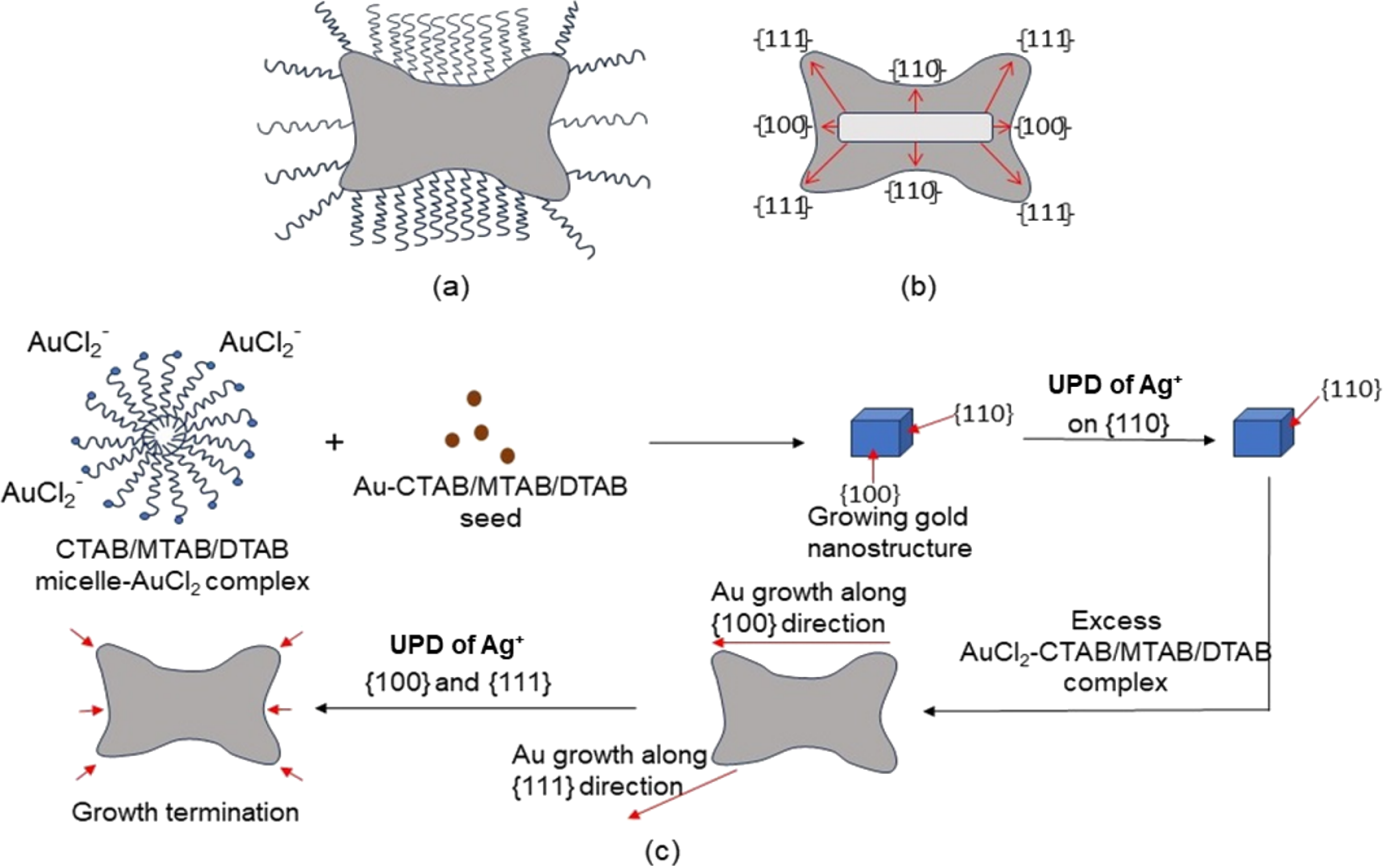
Growth mechanism of gold nanomakura particles (AuNM). (a) AuNM surrounded by surfactant micelles; (b) growth facet of AuNM; and (c) stepwise growth mechanism during AuNM formation.

Seed-mediated synthesis approach has been adopted in the present work which is quite well known to generate anisotropic nanomaterials. The mechanism mainly involves the arrangement of surfactant micelles which dictates anisotropy in nanoparticle during synthesis. A seed is a tiny crystal of ≈2–3 nm that initiates growth of a nanoparticle when introduced in a proper growth solution. The growth solution contains excess of gold ions along with a weak reducing agent and AgNO_3_. The excess gold is used as a reservoir for particle formation. On the other hand, AgNO_3_ plays a vital role through under potential deposition (UPD) by modulating the growth in a particular fashion. In Figure 6, it can be seen differential UPD of AgNO_3_ along with the micellar structure arrangement of the surfactant which led to the generation of nanomakura-shaped nanoparticles.

### Seed-mediated and seedless synthesis of gold nanorods (AuNRs)

**Figure 7:**
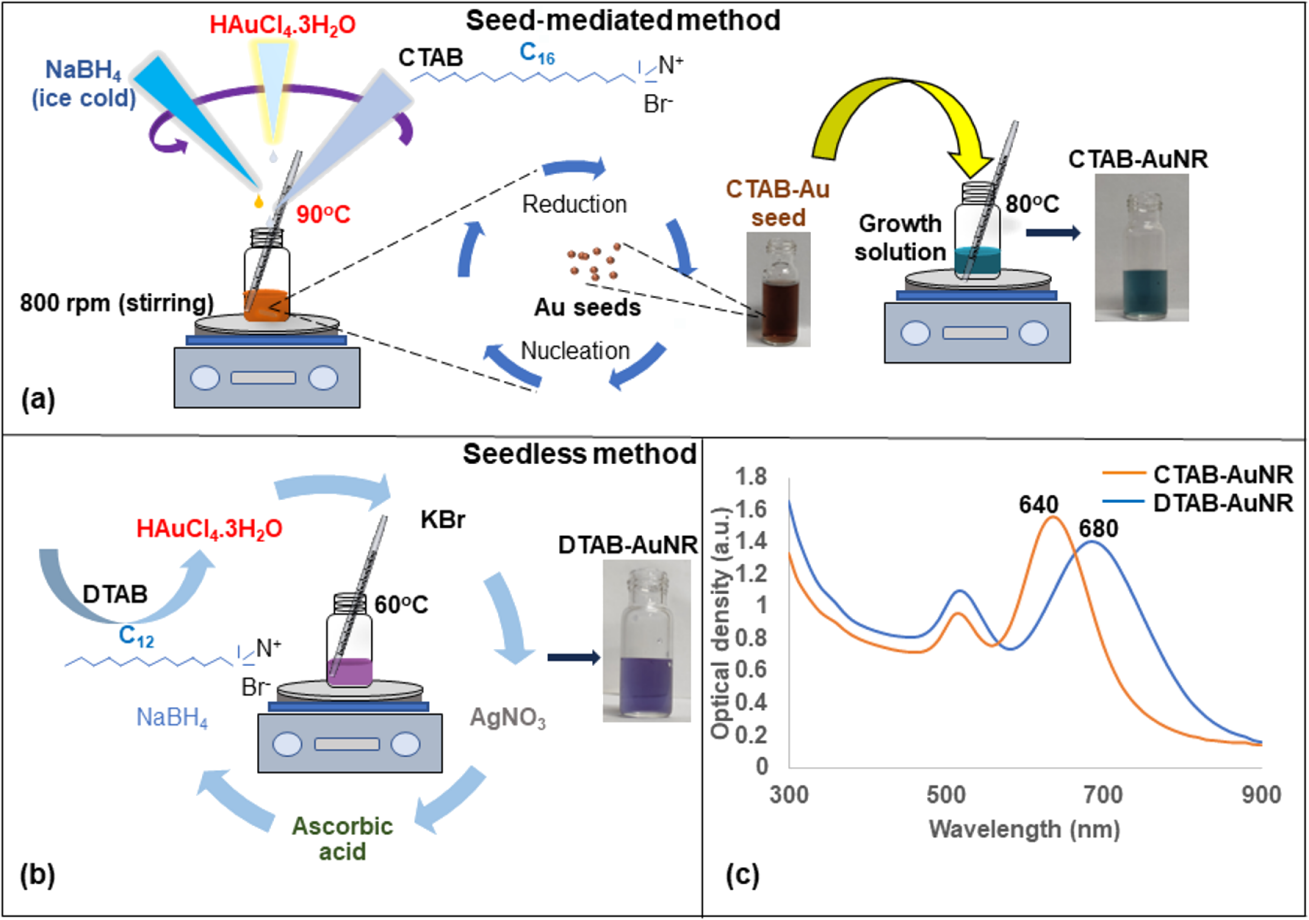
(a) Seed-mediated synthesis of CTAB-capped AuNR. (b) Seedless synthesis of DTAB-capped AuNR. (c) Absorption spectra of CTAB-AuNR and DTAB-AuNR, respectively, over a range of 300–900 nm.

In this experiment, two different approaches were adopted for synthesizing rod-shaped nanoparticles using CTAB and DTAB, respectively. Seed-mediated synthesis was adopted for synthesizing CTAB-capped AuNR, whereas the seedless method was used for synthesizing DTAB-capped AuNR. The sole purpose of the experiment was to obtain gold nanorods of longer and shorter lengths which is evident through the obtained absorption spectra as shown in Figure 7c. CTAB-AuNR and DTAB-AuNR showed LSPR at 640 and 680 nm, respectively. Furthermore, the synthesized AuNR were incorporated into k-CG hydrogels and checked for their absorption spectra stability as shown in Supporting Information File 1, Figure S2.

### Photothermal response study of AuNMs and AuNRs

**Figure 8:**
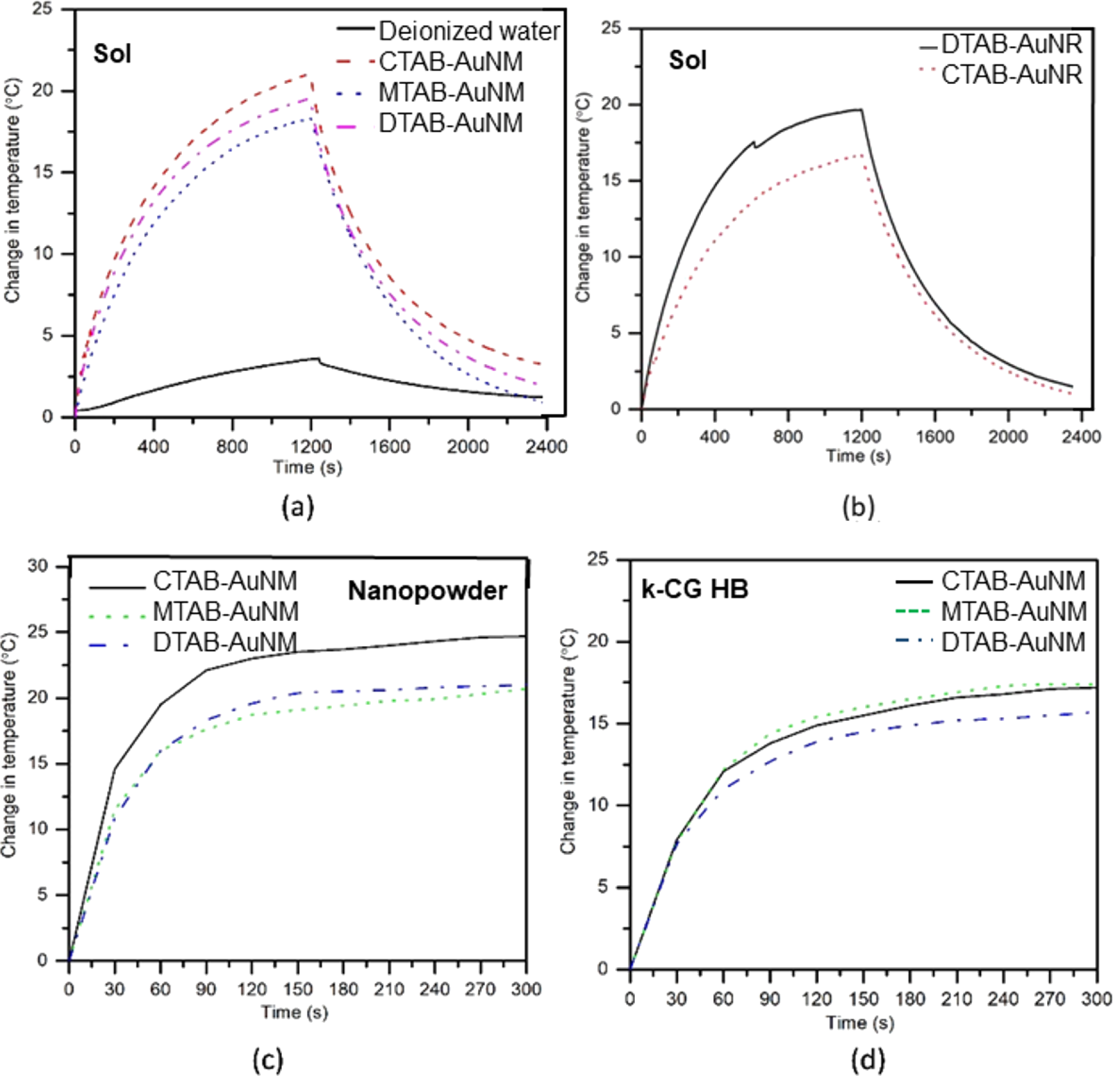
Anisotropic gold nanoparticles showing photothermal response of: (a) CTAB-AuNM, MTAB-AuNM, and DTAB-AuNM in colloidal state; (b) CTAB-AuNR and DTAB-AuNR in colloidal state; (c) CTAB-AuNM, MTAB-AuNM, and DTAB-AuNM in solid state (powder form); (d) CTAB-AuNM, MTAB-AuNM, and DTAB-AuNM after incorporation within k-CG hydrogel beads (HB) (powder form).

The measured photothermal response (i.e., temporal change in temperature) of various gold nanomakura particles and gold nanorods upon photothermal interaction in different forms (i.e., suspension, powder, and incorporated within k-CG hydrogels) is shown in Figure 8.

The net temperature rise of the gold nanomakura suspension and deionized water during visible broadband irradiation ON (heating) and OFF (cooling) for 1200 seconds each, measured using a “K-type” thermocouple is shown in Figure 8. Figure 8a shows the heating and cooling of gold nanoparticle suspensions of CTAB-AuNM, MTAB-AuNM, and DTAB-AuNM, respectively, whereas Figure 8b shows heating and cooling of DTAB-AuNR and CTAB-AuNR, respectively. From Figure 8a, it was observed that the temperature of deionized water reached up to ≈3 °C under irradiation with a light source. Also, suspensions of CTAB-AuNM, MTAB-AuNM, and DTAB-AuNM attained maximum temperature rise of up to 21.0, 18.3, and 19.5 °C on visible broadband interaction, respectively. On the other hand, CTAB-AuNR and DTAB-AuNR suspensions showed a temperature rise of up to 16.7 and 20 °C, respectively, as shown in Figure 8b. Further, in the OFF case (cooling), the exponential decay of the temperature in the nanoparticle suspension was observed.

The rise in temperature of the powdered nanoparticles and of nanoparticles incorporated in hydrogel beads during visible broadband irradiation in the ON case (heating) for 300 s was measured using a thermal camera (FLIR A655sc). The temperatures of the powdered CTAB-AuNM, MTAB-AuNM, DTAB-AuNM at time = 0 s (initial) and 300 s (at the end of irradiation) are shown in Figure 8a–c, respectively. The temporal variation in temperature of the powdered CTAB-AuNM, MTAB-AuNM, and DTAB-AuNM on photothermal interaction with a visible broadband light source for 300 s are shown in Figure 8c. The temperature rise in AuNMs capped with CTAB, MTAB, and DTAB attains up to ≈25, ≈21, and ≈21 °C, respectively, in powdered form. The thermal images captured for the same conditions are shown in Supporting Information File 1, Figure S3.

### Photothermal response of AuNRs in powder form

**Figure 9:**
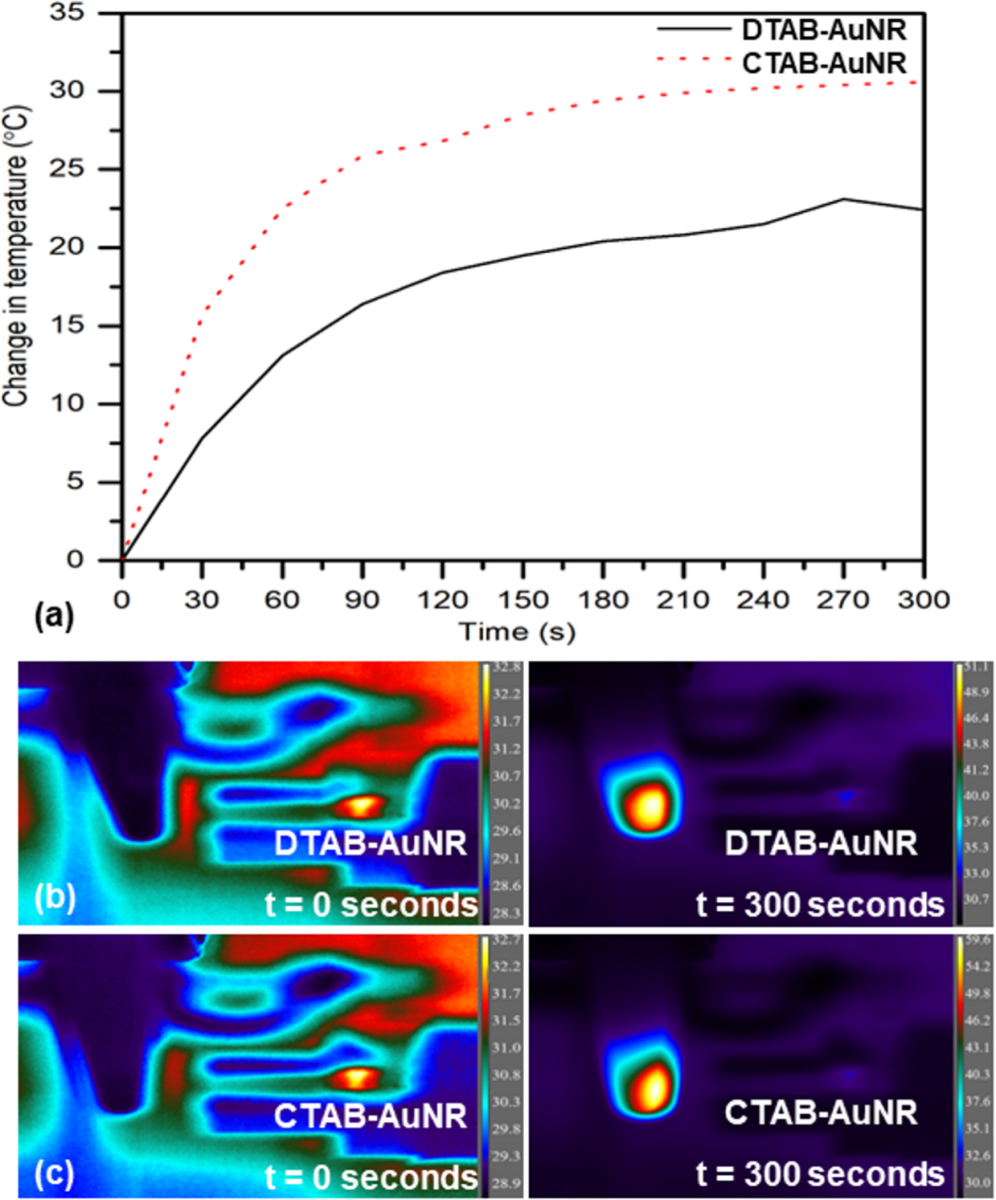
(a) Temporal variation in the corresponding temperatures on photothermal interaction with a visible broadband light source in DTAB- and CTAB-capped AuNR in powdered form. (b,c) Corresponding thermal images for temperature rise in AuNR capped with DTAB and CTAB, respectively, at time = 0 s (initial) and 300 s (after irradiation).

It was observed that DTAB-AuNR and CTAB-AuNR in powdered form attained up to 22.4 and 30.6 °C temperature rise upon interaction with the visible broadband light source as shown in Figure 9.

### Photothermal response of anisotropic nanoparticles in k-CG hydrogel beads

**Figure 10:**
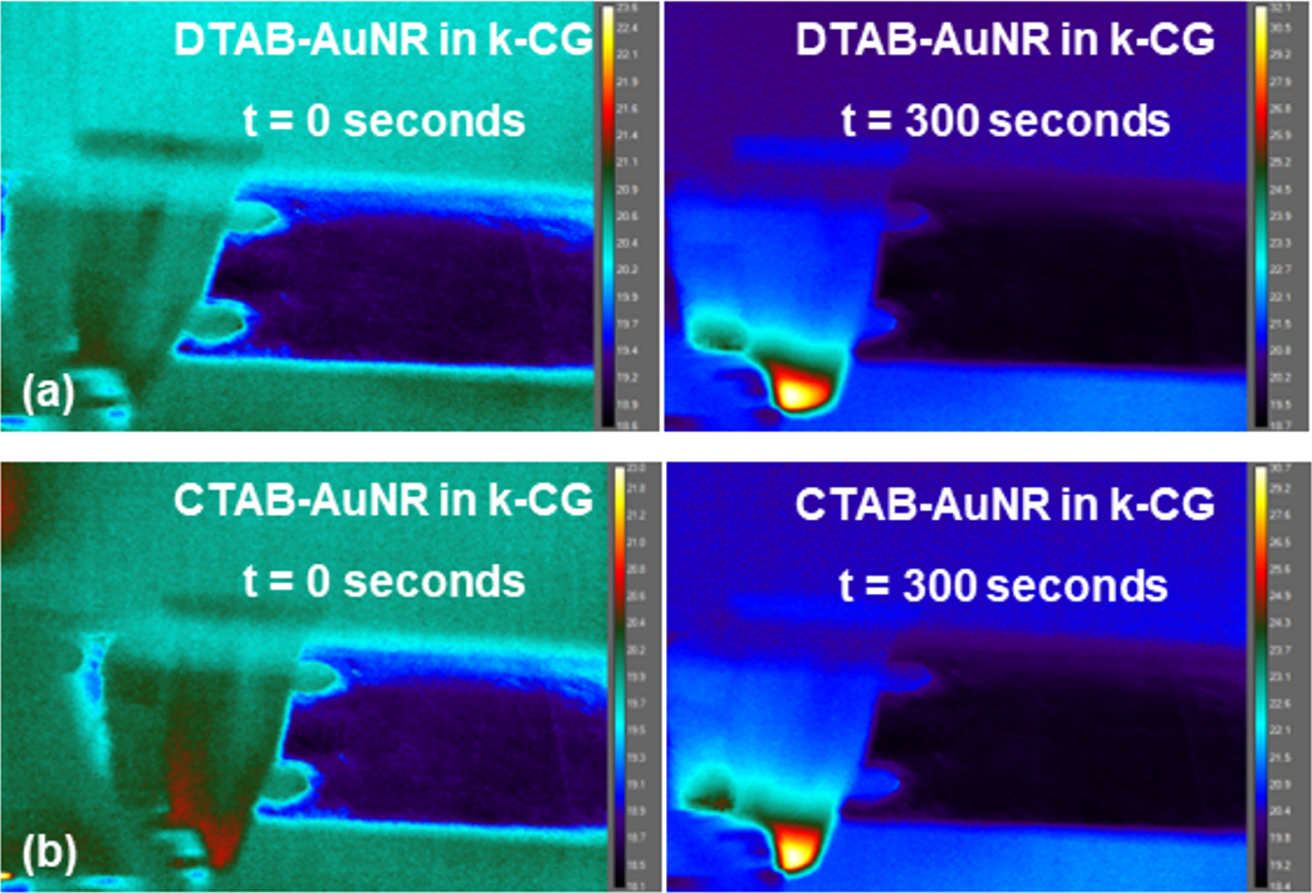
Thermal images showing photothermal response in (a) DTAB-AuNR and (b) CTAB-AuNR after incorporation into k-CG hydrogels at time = 0 s (initial), and 300 s (after irradiation).

The temperature of the nanoparticles (AuNMs) incorporated in hydrogel beads at time = 0 s (initial) and 300 s (after irradiation) is shown in Figure 10.

**Figure 11:**
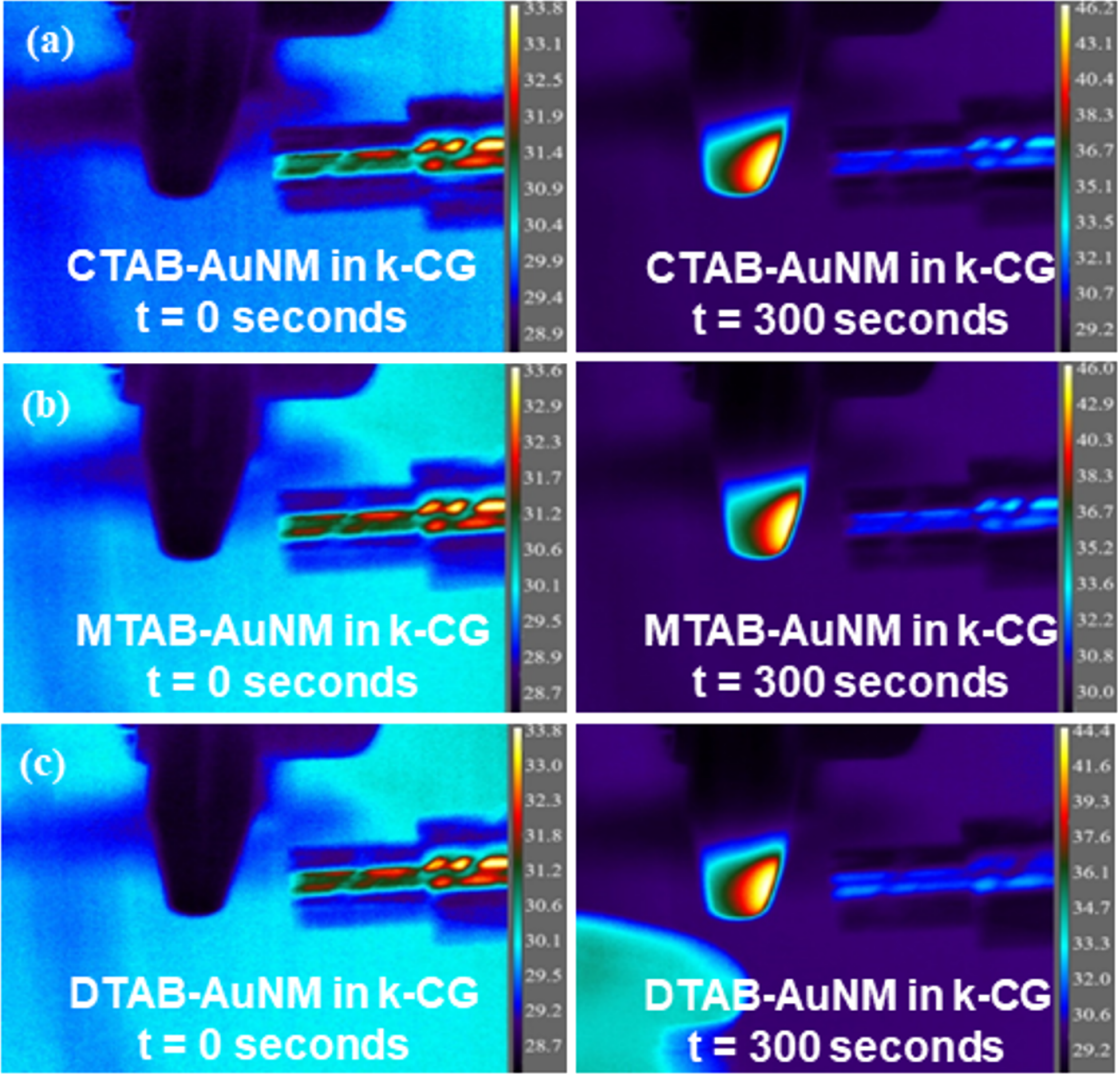
Thermal images showing photothermal response in (a) CTAB-AuNM, (b) MTAB-AuNM, and (c) DTAB-AuNM after incorporation into k-CG hydrogels at time = 0 s (initial) and 300 s (after irradiation).

Also, the temporal variation in the temperature of the nanoparticles in hydrogel beads was measured for 300 s upon irradiation. From Figure 8d and Figure 11, it was observed that the net temperature rise of the nanomakuras (CTAB-AuNM, MTAB-AuNM, and DTAB-AuNM) after incorporation into hydrogel beads could be attained up to ≈17.2, ≈17.2, and ≈15.7 °C, respectively, upon irradiation. The net temperature rise for DTAB-AuNR and CTAB-AuNR was attained up to ≈11.3 and ≈9.9 °C, respectively.

## Discussion

In this work, we adopted a seed-mediated approach to synthesize anisotropic nanostructures, viz., pillow shaped named as nanomakura (makura is a Japanese word meaning “pillow”). Surfactants belonging to the quaternary ammonium group of the same family (CTAB, MTAB, and DTAB) were used for synthesis. The anisotropic structure reported in this work resembles nanomakura gold nanoparticles as reported elsewhere for the first time [12]. However, the synthesis reported in this work is conspicuously different in terms of synthesis protocol and chemical constituents used. The main synthesis goal of this work was to study the effect of the carbon tail length of surfactants on the morphology of the synthesized nanostructures. Thus, keeping every parameter constant except the surfactant type during the reaction was paramount. The AgNO_3_ to ascorbic acid ratio was kept uniform in respective growth solutions containing CTAB, MTAB, and DTAB of the same concentration to facilitate longitudinal growth of the nanostructures. The disappearance of the yellow colour into a transparent growth solution upon introducing ascorbic acid indicated partial reduction of Au^3+^ to Au^+^, since ascorbic acid is a weak reducing agent. The introduction of seeds rapidly changed the colourless solution into blue, indicating complete reduction of Au^+^ to Au^0^. This was due to diffusion of Au^0^ atoms toward the {111} facet of the AuNMs, since mixed micelle structures are less densely packed at this facet as compared to that of the facets {110} and {100}, respectively [28]. Therefore, the templates favoured longitudinal growth curving outwards, giving the shape of nanomakura. The shape of nanomakura was visualized and confirmed using TEM and AFM. Figure 4a–c showed TEM images of nanoparticles confirming the nanomakura shape. However, it was interesting to notice that the nanomakura shape is more pronounced in DTAB-AuNM. This was also confirmed using AFM considering the AuNMs under a three-dimensional view. Furthermore, 3D images were captured using AFM as shown in Figure 4d–f confirming the topography of AuNMs. Therefore, it was clearly observed from the complementing results obtained through TEM and AFM that the shape of the nanoparticles is makura. It can be assumed that the breaking of nanorod-shape symmetry into a nanomakura shape was determined by the surfactant capping, clearly indicating the effect of carbon tail length. The breaking of symmetry was due to a gradual decrease in micelle density on the edges giving the shape of a pillow as shown in Figure 6 The highest intensity of {111} observed in the XRD diffractogram (Figure 5b) also confirmed the preferred longitudinal growth towards the {111} facet. The XRD results as shown in Figure 5b along with SEM/EDX results (Supporting Information File 1, Figure S4) confirmed that the AuNMs were made of gold. The formation of bilayer assembly of surfactants was confirmed through FTIR spectroscopy. C–H scissoring vibrations of H_3_C–N^+^ and C–N^+^ stretching are relatively less intense and slightly shifted in surfactant-capped gold nanoparticles with an appearance of a single band at 669 cm^−1^ as shown in Figure 5a. This is clear proof of the containments that surfactant alkyl chains are subjected to, resulting in the formation of a compact layered structure around the nanoparticle [25]. Also, it was interesting to observe the differences in aspect ratios of nanomakuras capped with CTAB, MTAB, and DTAB respectively. Nanomakuras capped with CTAB showed the highest aspect ratio, which was followed by MTAB- and DTAB-capped AuNM, respectively, as shown in Table 2. The LSPR of CTAB-AuNM, MTAB-AuNM, and DTAB-AuNM at 670, 650, and 630 nm, respectively, corroborated the aspect ratio results. This indicated that LSPR and aspect ratio are directly proportional to each other. Finally, the decreasing trend in LSPR and aspect ratio as shown in Figure 1c and Table 2 can be attributed to the carbon tail length of the respective surfactants: CTAB, MTAB, and DTAB containing 16, 14, and 12 carbon atoms, respectively. Since carbon chain length dictates the compactness of mixed micelle structures, micelles with longer carbon tail lengths form more compact bilayer structures and vice versa. Also, the density of mixed micellar structure of CTAB is higher and is followed by MTAB and DTAB, respectively; thus, resulting in the formation of AuNMs of different aspect ratios. The corroboration of results obtained through optical spectroscopy, aspect ratio calculation, FTIR, and XRD measurements clearly indicated the profound effect of carbon tail length on the morphology of AuNMs.

The synthesized nanomakuras, due to their intrinsic properties and optical activity close to the NIR region, have the potential to exhibit photothermal conversion which has been manifested for their application in minimal-invasive photothermal therapy (PTT). The photothermal conversion is known to be significantly governed by LSPR where the state/milieu of the nanoparticles can play an important role [18]. Significant advancements have been achieved in using soft/smart materials such as hydrogels in photothermal therapy due to their thermoresponsive behaviour. Kappa-carrageenan is a natural oligosaccharide known for its thermoresponsive behaviour making it a suitable candidate for photothermal conversion. Therefore, it was necessary to check the stability of AuNMs in k-CG hydrogels which was confirmed upon exhibition of plasmon peaks at 600–700 nm wavelengths as shown in Figure 3. The gold nanoparticles in association with hydrogels upon exposure to a broadband light source of 400–700 nm converted light energy into heat, thus, stimulating thermal response. The photothermal response of AuNMs was investigated for different state viz., colloidal and solid states (powder/hydrogel beads). Figure 8a, c, and d showed photothermal conversion of AuNMs in terms of temperature rise at colloidal and solid states, respectively. In the colloidal form, the rise of temperature in CTAB-AuNM, MTAB-AuNM, and DTAB-AuNM was 21.0, 18.3, and 19.5 °C respectively. This clearly indicated a slight impact of LSPR on photothermal conversion ability where LSPR at a higher wavelength was responsible for higher photothermal response and vice versa. To check whether the temperature rise was solely due to LSPR, we synthesized CTAB-AuNR (λ_max_640) of shorter length and DTAB-AuNR (λ_max_680) of longer length opting out the middle wavelength (i.e., MTAB-AuNR) as shown in Figure 7. Since AuNRs are conventionally used nanostructures in photothermal conversion studies, we chose one shorter and one longer nanorod to make the current study conclusive [29]. Interestingly, CTAB-AuNR and DTAB-AuNR showed a significant temperature rise of 16.7 and 20 °C, respectively, as shown in Figure 8b at colloidal state. The high temperature change in case of DTAB-AuNR clearly showed the proof of containment of LSPR in determining photothermal response. Therefore, the difference in the rise of temperature between nanomakura and nanorods can be assumed due to their difference in NIR absorption peak (i.e., LSPR only) irrespective of the surfactant capping. The results can be further corroborated by the effect of carbon tail length, which dictated linear dimension, profoundly impacting their photothermal conversion ability. Furthermore, when in k-CG hydrogel as shown in Figure 11, the photothermal conversion ability through temperature rise of AuNMs corroborated the results obtained in the colloidal form, indicating excellent stability of AuNMs in terms of photothermal conversion ability. The temperature rise of ≈17.2, ≈17.2, and ≈15.7 °C in AuNMs capped with CTAB, MTAB, and DTAB, respectively, in k-CG clearly showed insignificant attenuation of temperature in k-CG hydrogels. Therefore, it can be concluded that the rise in temperature was solely due to the presence of AuNMs. This was further validated by measuring the photothermal response in k-CG hydrogel beads without any nanoparticle incorporation as shown in Supporting Information File 1, Figure S5. On the other hand, AuNRs capped with CTAB and DTAB showed a temporal rise in temperature of ≈16.7 and ≈20.0 °C in the colloidal form. However, upon incorporation into k-CG hydrogels, the temporal temperature rise was ≈9.9 and ≈11.3 °C, respectively. This could be attributed to the morphology and assembly of the nanostructures within the gel matrix since the concentration of AuNMs and AuNRs incorporated into the k-CG hydrogels was the same. Therefore, it can be assumed that AuNMs due to their outward concave growth at the edges provided more surface area and greater stability in k-CG hydrogels, leading to greater photothermal response as compared to that of AuNRs. The intactness and ability of AuNMs to exhibit photothermal conversion even after incorporation into k-CG hydrogels show a possible use of the designed nanostructures for therapeutic purposes in the wavelength window of 600–700 nm [30].

## Conclusion

The synthesized pillow-shaped nanostructures, otherwise called nanomakura, exhibited a multimodal absorption peak spectrum in the 600–700 nm wavelength region. CTAB-AuNM, MTAB-AuNM, and DTAB-AuNM showed NIR absorption peak wavelengths at 670, 650, and 630 nm, respectively. The different absorption peak wavelengths of CTAB-AuNM, MTAB-AuNM, and DTAB-AuNM were influenced by the number of carbon atoms present in each surfactant. The effect of carbon tail length dominated in a linear growth fashion during nanoparticles synthesis, directing LSPR and aspect ratio of NMs. The physicochemical properties of the designed nanomakuras were validated using XRD and FTIR. Their excellent colloidal stability was confirmed through absorption spectral studies in different media. Also, the optical absorption spectra of AuNMs were retained when incorporated into k-CG hydrogels showing their robust nature. Furthermore, these nanoparticles before and after their incorporation into k-CG were checked for photothermal response. On the contrary, AuNRs after incorporation into k-CG hydrogels showed a small increase in temperature as compared to that of AuNMs upon laser irradiation. Therefore, the intactness and ability of AuNMs as compared to AuNRs in exhibiting photothermal response within k-CG hydrogel beads show a possible use of the designed nanostructures for biomedical soft materials operating within the 600–700 nm window. However, a future study of the usage of these materials in biological systems is needed for exploiting their true potential application.

## Experimental

### Materials

All the following chemicals were used as received: hexadecyltrimethylammonium bromide (Fluka), myristyltrimethylammonium bromide (Fluka), dodecyltrimethylammonium bromide (TCI Chemicals), chloroauric acid (HAuCl_4_·3H_2_O, Finar Chemicals), silver nitrate (AgNO_3_, Merck), ʟ-(+)-ascorbic acid (Sigma-Aldrich), and sodium borohydride (NaBH_4_, Sigma-Aldrich). Prior to synthesis, all the glassware was cleaned with aqua regia and further rinsed with double-distilled (DD) water. Double-distilled water was used throughout the experiments.

### Methodology

#### Synthesis of makura-shaped gold nanoparticles

**Step 1: Synthesis of surfactant-capped gold seeds.** Individual surfactant-capped seeds were prepared for the synthesis of different surfactant-capped gold nanomakura. Initially, surfactant-capped gold seeds were synthesized using the available surfactants (CTAB, MTAB, and DTAB) via a modified protocol of Murphy et al. (2003) [24]. An amount of 200 μL of a 0.025 M HAuCl_4_·3H_2_O solution was added to 2 mL of 0.1 M surfactant solutions (CTAB, MTAB, and DTAB respectively) and vigorously stirred at 700 rpm at 90 °C. Finally, 800 μL of freshly prepared ice-cold NaBH_4_ (0.01 M) was added, which turned the solution brownish in colour. The appearance of a brownish colour confirmed the reduction of Au^3+^ to Au^0^.

**Step 2: Growth-mediated synthesis of gold nanomakura.** In brief, a protocol that combines seed-mediated growth with controlled particle etching was used. The reaction parameters were kept uniform throughout the three growth solutions. Initially, the surfactant stock solutions of 0.1 M (CTAB, MTAB, and DTAB) were heated, and 15 mL of the respective solutions were transferred into 50 mL centrifuge tubes. The temperature of the surfactant solutions measured with a thermometer was 80 °C. Subsequently, 1 mL of 0.01 M HAuCl_4_·3H_2_O was added to each surfactant solution, which was followed by the addition of 100 μL of 0.01 M AgNO_3_. The solution appeared slightly orange/yellowish in colour. Next, 100 μL of 0.2 M ascorbic acid was added and gently swirled, turning the solution colourless. This is due to the change in the oxidation state of gold ions. Finally, 25 μL of the seeds prepared in Step 1 was added to their respective surfactant growth solutions and was left undisturbed for 15 min. The colour of the samples appeared blue indicating the formation of anisotropic gold nanoparticles. Finally, the synthesized AuNMs were centrifuged at 10,000*g* for 15 min.

#### Synthesis of gold nanorods capped with CTAB and DTAB

The synthesis of gold nanorods capped with CTAB and DTAB followed seed-mediated and seedless-mediated synthesis respectively. For synthesizing CTAB-AuNR, 5 mL of 0.1 M CTAB was used which was raised to 80 °C initially. After reaching the temperature, 500 uL of 0.01 M HAuCl_4_·3H_2_O was added. This was followed by the addition of 50 μL 0.01 M AgNO_3_. Subsequently, 200 μL of 1 M HCl was added which was followed by the addition of 80 μL ascorbic acid of 0.1 M. Finally, 50 μL of seed solution was added to the growth solution. The whole process of synthesis was carried at 80 °C. The synthesized CTAB-AuNR were centrifuged at 16,000*g* for 15 min.

Seedless synthesis of DTAB-capped gold nanorods (DTAB-AuNR) started with 5 mL of 0.1 M DTAB which was raised to 60 °C initially and then followed by the addition of 500 μL of 0.01 M HAuCl_4_·3H_2_O. Further, 200 μL KBr of 0.02 M was added. Subsequently, 50 μL AgNO_3_ of 0.01 M was added and was followed by the addition of 80 µL of 0.1 M ascorbic acid. Finally, 50 μL of 1 mM NaBH_4_ was added to complete the reaction. The synthesis was carried out at 60 °C. The synthesized DTAB-AuNR were centrifuged at 10,000*g* for 15 min.

#### Incorporation of gold nanoparticles into k-carrageenan hydrogels

A stock solution of k-carrageenan was prepared by mixing 1 g k-CG in 40 mL of DD water, and heated until the temperature of the solution raised to 80 °C. The solution was kept under constant stirring at 800 rpm on a hot plate for complete dissolution. After the complete dissolution of k-CG, 7 mL of the stock solution was transferred to a 15 mL glass vial into which 1 mL of purified gold nanoparticles was dispersed. It is to be noted that a fixed number of particles per 1 mL has been used to maintain the reproducibility of particle density in the hydrogel. A volume of 1 mL of the sample showing 4 OD was achieved by pooling the required number of pellets, and was dispersed into 7 mL of k-carrageenan making the solution blue in colour. Finally, the solution was drop cast in a KCl solution (1 M) using a 2.5 mL syringe. This resulted in the formation of bright blue coloured spherical k-CG hydrogel beads incorporated with gold nanoparticles. The beads were further rinsed with DD water, subsequently washed with 70% ethanol, and finally dried in a hot air oven for 3 h at 60 °C. The dried beads were used for the subsequent experiments and characterization techniques.

### Characterization techniques

The absorption spectra of the synthesized gold nanoparticles capped with CTAB, MTAB, and DTAB were measured using an Epoch2 (BioTek, USA) spectrophotometer. An amount of 1 mL of each sample after one wash was taken into consideration for the measurement. The absorbance range was set to 300–900 nm during all measurements. The zeta potential measurement was performed using a Zetasizer (Nano ZS, Malvern UK). The sample was transferred into a DTS1070 cuvette and the instrument was run for the zeta potential measurement. The actual size, morphology, and aspect ratio calculation of the synthesized nanoparticles were observed under a transmission electron microscope (120 kV, Tecnai, FEI, The Netherlands) at multiple regions. An atomic force microscope (Witec Alpha 300RA) equipped with a solid-state laser operating at 532 nm with a power of 0.7 mW, a grating of 600 L/mm, and an objective lens of 50× (Zeiss) was used in noncontact (tapping) mode. The cantilevers of length 125 μm and width 4 μm were used at a resonance frequency of 142 Hz and at a constant force of 42 N/m. The sample was prepared by loading it on mica coverslips which was subsequently fixed onto glass slides and air dried for an hour before the measurements. The EDX measurements were performed using a scanning electron microscope (Quanta 450 FEG, FEI, The Netherlands). The crystalline structure of gold nanoparticles in CTAB-, MTAB-, and DTAB-capped nanomakura was analysed through a Panalytical XRD machine. For this, 30 mg of the powdered sample was used to completely cover the holder and was exposed to X-rays for the measurement. Surface functionalization validation of CTAB-, MTAB-, and DTAB-capped nanomakura was assessed through FTIR spectroscopy. For this, 1–2 mg of the dried form of the nanoparticles and beads incorporated with nanoparticles for each sample was placed on a diamond point of FTIR (Thermo Scientific, Nicolet iS5, USA) using the software OMNIC™ for the measurements over a transmittance spectra range of 500–4000 cm^−1^.

### Photothermal response of the synthesized AuNMs

#### Experimental setup for the photothermal response measurement of AuNMs suspensions

In order to conduct photothermal studies of the synthesized AuNMs suspensions, 1.5 mL of various gold nanoparticle suspensions (OD = 1) was taken in a quartz cuvette of 10 mm path length. The photothermal studies were performed by using a broadband light source (400–700 nm) with an intensity of ≈0.5 W/cm^2^ (beam diameter ≈13 mm). A thermopile detector (919P-250-35, Newport Corporation) and a digital power meter (843-R, Newport Corporation) were used to measure the optical power of the incident radiation. The net temperature rise of these AuNM suspensions was measured using a "K-type" thermocouple, and data were collected by using a data acquisition card (NI-myDAQ 9214 from National Instruments). The temperature increase and decrease of the suspension were measured for 20 min each.

#### Experimental setup for photothermal response measurement of AuNMs incorporated gel beads

For the temperature increase measurement of AuNMs incorporated gel bead samples (powder form), the experiments were performed by using a 20 mg powder placed in 1.5 mL conical centrifuge tubes. The photothermal studies were performed by using a broadband light source (400–700 nm) with an intensity of ≈0.5 W/cm^2^ (beam diameter ≈13 mm). In this case, the temperature increase was measured as thermal images acquired using an infrared thermal camera (FLIR, A655Sc).

## Supporting Information

File 1 Additional figures.

## Data Availability

All data that supports the findings of this study is available in the published article and/or the supporting information to this article.
