# Supplementary material for: Gold nanomakura: nanoarchitectonics and their photothermal response in association with carrageenan hydrogels
Source: Beilstein J Nanotechnol. 2024 Jun 7;15:678–93. doi: 10.3762/bjnano.15.56 (PMC11181249; doi:10.3762/bjnano.15.56)
Supplement: File 1 — Additional figures. [file Beilstein_J_Nanotechnol-15-678-s001.pdf]

BEILSTEIN JOURNAL OF NANOTECHNOLOGY

Supporting Information

for

Gold nanomakura: nanoarchitectonics and their photothermal response in association with carrageenan hydrogels

Nabojit Das, Vikas, Akash Kumar, Sanjeev Soni and Raja Gopal Rayavarapu

Beilstein J. Nanotechnol. **2024,** *15,* 678–693. doi:10.3762/bjnano.15.56

Additional figures

License and Terms: This is a supporting information file under the terms of the Creative Commons Attribution License (https://creativecommons.org/licenses/by/4.0). Please note that the reuse, redistribution and reproduction in particular requires that the author(s) and source are credited and that individual graphics may be subject to special legal provisions.
The license is subject to the *Beilstein Journal of Nanotechnology* terms and conditions: (https://www.beilstein-journals.org/bjnano/terms)

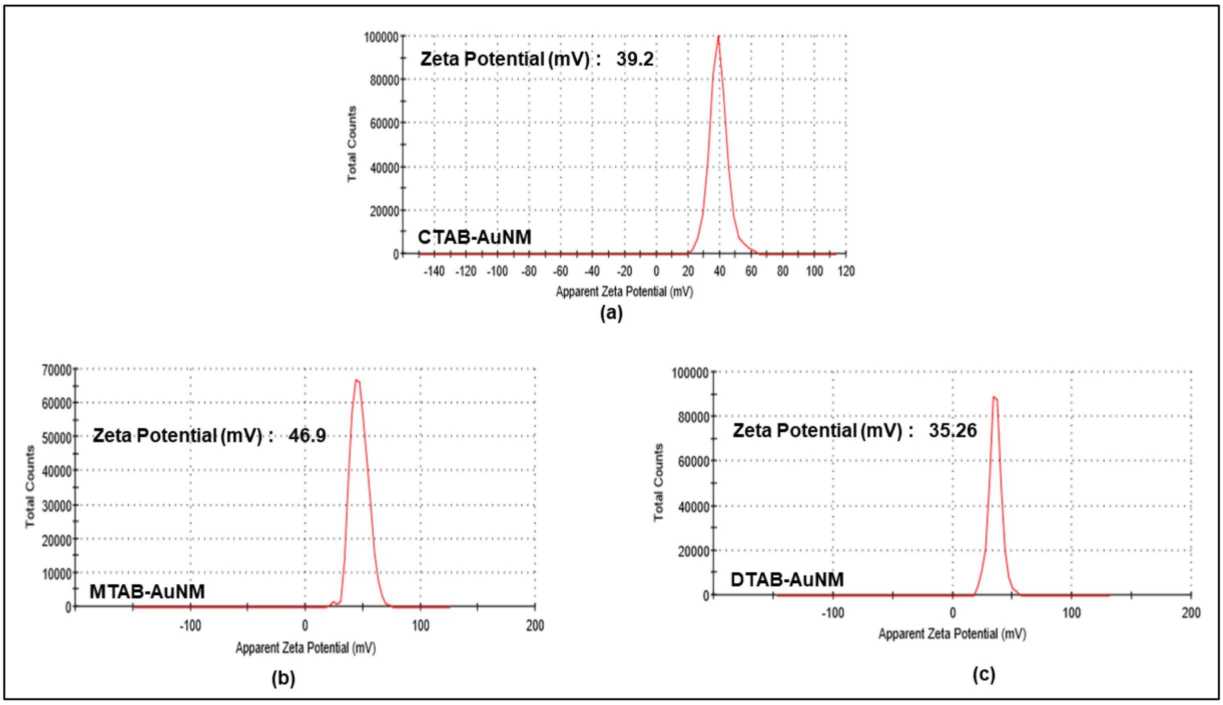

Figure S1: Zeta potential value of CTAB-AuNM, MTAB-AuNM, and DTAB-AuNM, respectively.

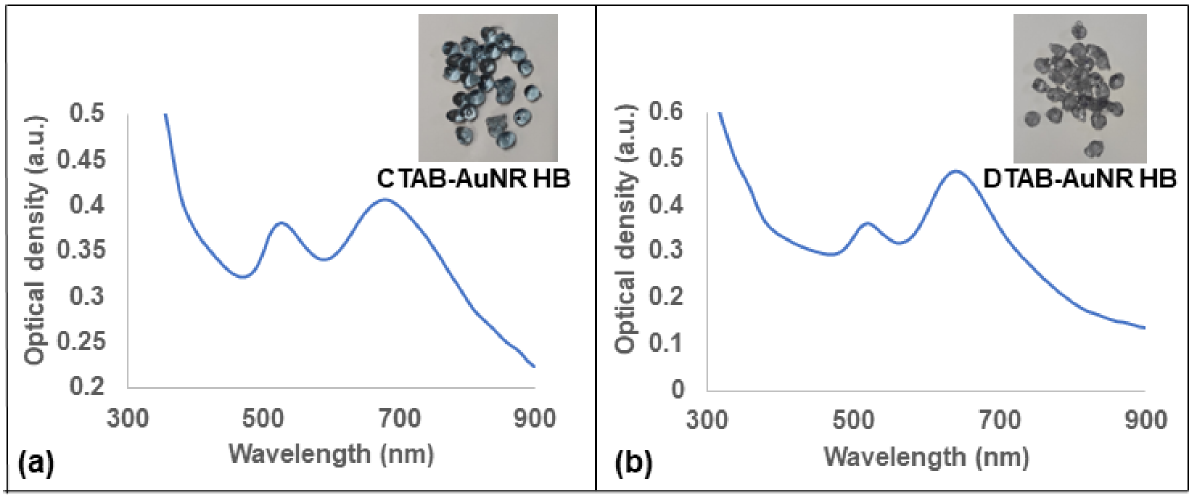

Figure S2: Absorption spectra of CTAB-AuNR and DTAB-AuNR after their incorporation in k-CG. The inset images show the hydrogel beads after casting in KCl.

Photothermal interaction of anisotropic gold nanoparticles in solid state (powder form and hydrogel beads)

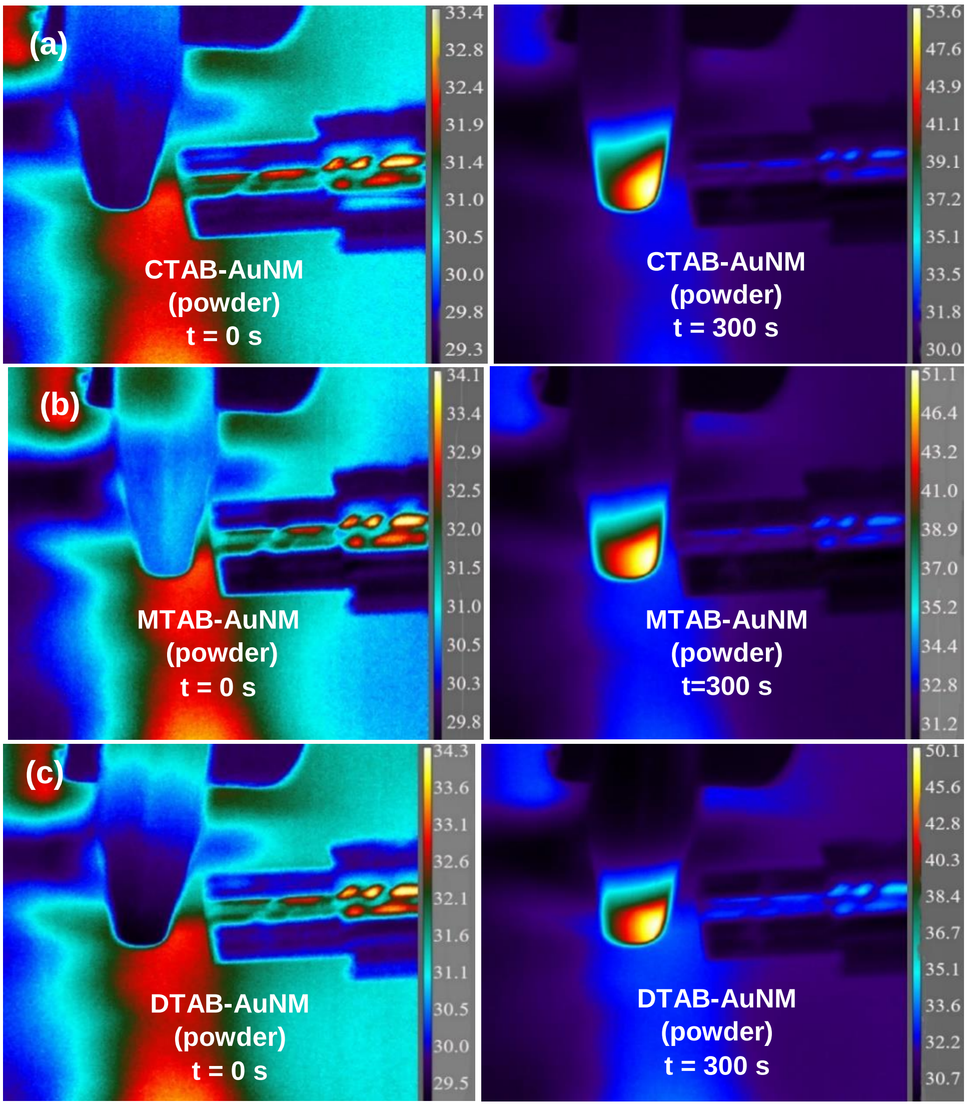

Figure S3: Photothermal response of gold nanomakura in powdered form: (a) CTAB-AuNM, (b) MTAB-AuNM, (c) DTAB-AuNM) at time = 0 s (initial), and 300 s (at the end of irradiation).

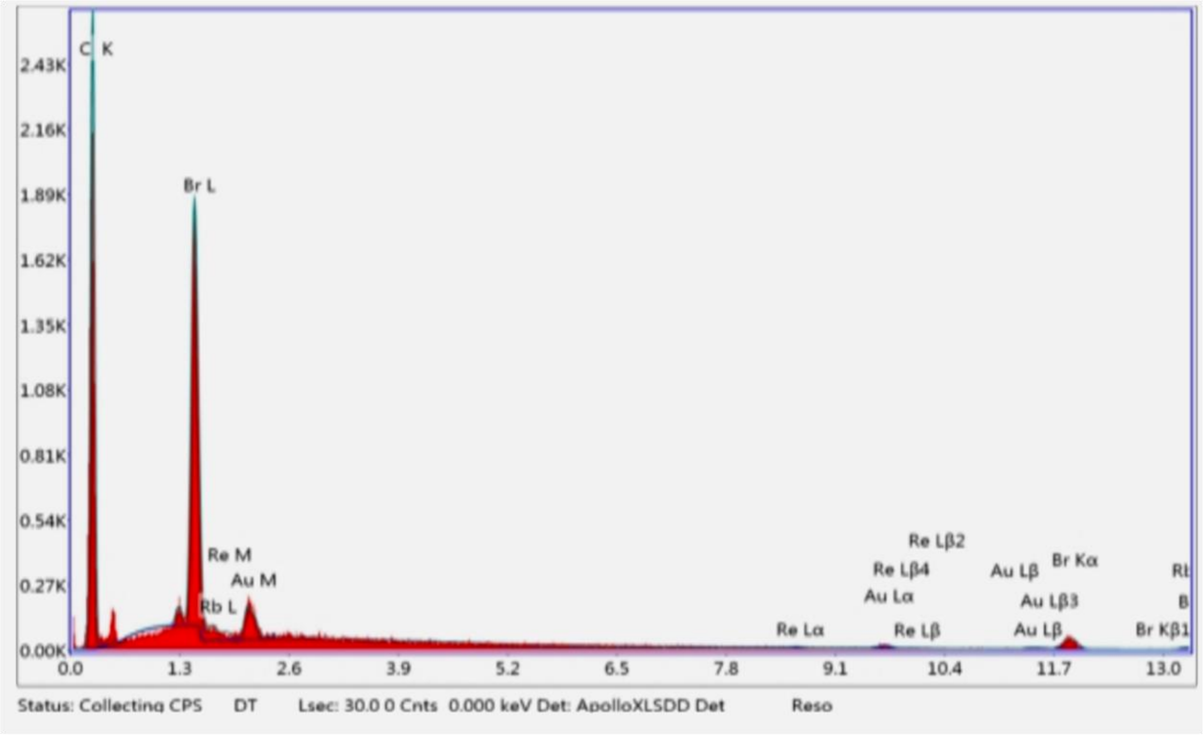

Figure S4: A typical SEM EDX spectrum confirming the presence of gold in AuNM.

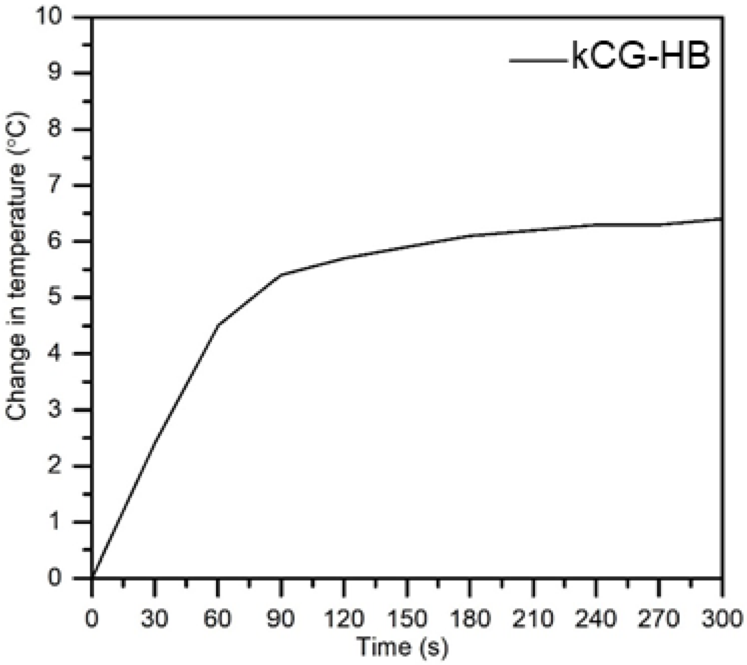

Figure S5: Temporal variation in temperature of the hydrogel beads powder without nanoparticles on photothermal interaction with a visible broadband light source.
